# Comparative Impacts of Conventional and Biodegradable Microplastics on Boscalid Behavior and Toxicity in Soil–Earthworm System

**DOI:** 10.3390/molecules31132268

**Published:** 2026-06-29

**Authors:** Shihang Han, Jiyan Miao, Wei Sun, Xinrui Dang, Qi Chen, Xiaoxuan Sun, Yifan Yue, Jinling Diao, Wentao Zhu

**Affiliations:** Innovation Center of Pesticide Research, Department of Applied Chemistry, College of Science, China Agricultural University, Beijing 100193, China; hsh18510919211@outlook.com (S.H.); zlwdmiao@126.com (J.M.); 13280701728@163.com (W.S.); swucq666@163.com (Q.C.); lingyinzi1201@gmail.com (J.D.)

**Keywords:** microplastics, boscalid, co-exposure, intestinal barrier, metabolomics

## Abstract

The widespread environmental presence of microplastics has led to their increasing co-occurrence with pesticides in agricultural soils, which raises concerns about their potential combined effects on pollutant behavior and toxicity. In this study, we investigated the environmental fate of boscalid and its toxicity to earthworms under co-exposure with two types of microplastics. Both polyethylene microplastics (PE) and polylactic acid microplastics (PLA) significantly enhanced boscalid retention in soil and delayed its degradation. Co-exposure impaired intestinal barrier function, promoted boscalid bioaccumulation, and triggered more severe oxidative stress and metabolic disturbances in earthworms. Notably, differences were observed between PE and PLA in their effects on boscalid behavior and earthworm responses. Our study suggests that microplastics may influence the ecological risk of boscalid through potential carrier effects and biological interface interactions and indicates mechanistic differences between conventional and biodegradable microplastics in modulating pesticide toxicity. These findings offer new insights into the environmental risk assessment of combined pollution.

## 1. Introduction

Defined as plastic fragments ≤ 5 mm, microplastics represent a diverse and globally significant class of pollutants [1]. Their main sources include personal care products, industrial waste, and textiles, whose materials fragment and persist in the environment [2]. Recent studies have revealed significant microplastic contamination in agricultural soils. For instance, the average abundance reached 9.8 × 10^3^ particles per kg of dry soil (Ds) in Yunnan Province, China [3], while agricultural soils in the Hangzhou Bay area have an average microplastic abundance of 521 particles/kg Ds [4]. High concentrations of microplastics in soils can alter soil physical properties and structure [5] and may affect the environmental behavior of other pollutants. Notably, polyvinyl chloride and polyethylene (PE) microplastics increase the adsorption of DDT and phenanthrene [6], and PE microplastics has been reported to promote chlorpyrifos migration into soils [7], thereby posing potential environmental risks. Moreover, microplastics have been found at varying levels in agricultural and sideline products [8], and their presence has even been reported in human excreta [9], highlighting their widespread occurrence and potential health implications.

Previous studies have reported that microplastics can damage the intestinal barrier of organisms to a certain extent [10] and serve as effective carriers of other pollutants. Such barrier disruption may facilitate the entry and accumulation of microplastics and associated pollutants in biological systems [11]. Beyond their carrier role, microplastics themselves may exert toxic effects and influence the toxicity of co-occurring pollutants [12]. For example, PE microplastics attenuate the inhibitory effect of Cu^2+^ on microalgae growth [13], whereas co-exposure to bisphenol A and PE microplastics has been shown to cause neurotoxicity in adult zebrafish, mainly through the MAPK pathway and calcium ion channels [14]. These findings underscore the importance of systematically investigating the combined effects of microplastics and pesticides on soil organisms.

To address microplastic pollution, the agricultural sector has begun adopting biodegradable plastics, such as polylactic acid (PLA) and polyhydroxyalkanoates [15]. However, biodegradable plastics do not completely eliminate the hazards posed by conventional microplastics. Because these materials are biodegradable, they can break down more quickly and extensively into microplastics, which may pose additional risks to soil ecosystems [16]. For example, PLA has been shown to alter the composition and functional activity of soil bacterial communities, potentially affecting soil fertility and structural properties [17]. PLA also exhibit toxicity to soil fauna comparable to that of PE. For instance, *Eisenia fetida* can accumulate PLA, leading to reduced reproductive capacity [18]. Exposure to both PE and PLA microplastics has been observed to similarly affect biomarkers associated with oxidative stress, neurotoxicity, and detoxification in *Eisenia fetida* [19]. Beyond their intrinsic toxicity, PLA and PE exhibit distinct interactions with co-existing pollutants. PLA possess stronger metal ion adsorption capabilities [20], and have been shown to more significantly enhance the toxicity of decabromodiphenyl ether to earthworm gut microbiota and disrupt the soil C/N cycling [21]. At present, most research has focused on conventional plastics, whereas studies on biodegradable microplastics remain limited due to their lower production volume. This may present a potential environmental threat to the sustainable development of soil ecosystems.

Boscalid is a widely used SDHI (succinate dehydrogenase inhibitor) fungicide. However, due to its extensive use, boscalid frequently migrates into soil and subsequently enters surface and groundwater systems, posing risks to ecosystems [22]. Reports have reported that boscalid concentrations in surface and groundwater in the U.S. can reach 2–3 μg/L [23], while Chinese agricultural soils have been found to contain significantly higher levels, up to 4.5 mg/kg [24].

The present study addresses the combined effects of conventional (PE) and biodegradable (PLA) microplastics with the pesticide boscalid in soil ecosystems. Previous studies have mainly focused on single microplastics or pesticides, or simple binary combinations. In contrast, our study compares different types of microplastics under both soil-only and earthworm–soil microcosm conditions. We evaluate the effects of PE and PLA on boscalid dissipation and earthworm responses. This provides a direct comparison between conventional and biodegradable microplastics in a soil system with biological relevance. The results suggest that co-exposure to PE or PLA with boscalid may increase ecological risk, which helps improve the understanding of their environmental impacts.

## 2. Results

### 2.1. The Effect of Combined Exposure of Boscalid and/or PE/PLA on the Environmental Behavior of Boscalid

**Figure 1:**
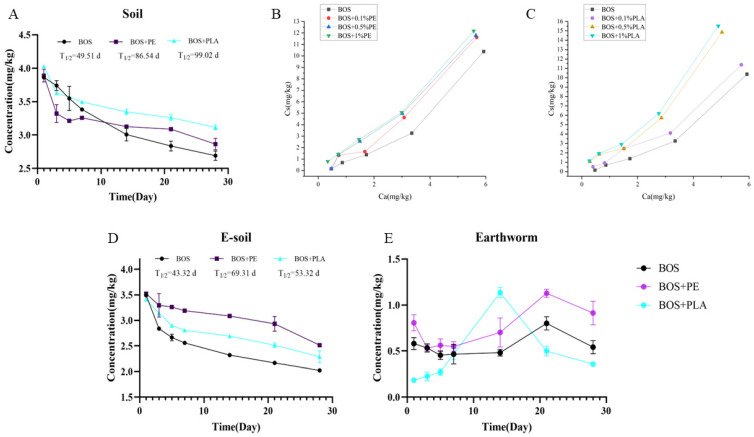
Environmental behavior and bioaccumulation of BOS in soil and earthworms. (**A**) Degradation dynamics of BOS in soil with or without PE/PLA. (**B**) Adsorption behavior of BOS coexisting with PE. (**C**) Adsorption behavior of BOS coexisting with PLA. (**D**) Degradation dynamics of BOS in the soil of the earthworm–soil microcosms with or without PE/PLA. (**E**) Bioavailability of BOS in earthworms. Data are presented as mean ± SD (*n* = 3 beakers/tubes).

The degradation behavior of boscalid in soil was first evaluated in the presence of PE and PLA. During the 28-day experimental period, boscalid concentrations in soil steadily decreased; however, the presence of PE or PLA markedly slowed its degradation rate. Based on first-order kinetic fitting of the observation data of 28 days, the half-life of boscalid in soil was estimated to be 49.51 days, whereas the estimated values increased to 86.54 days with PE and 99.02 days with PLA. As these estimated half-lives exceeded the exposure period, they should be interpreted as comparative indicators of relative persistence among treatments rather than directly observed degradation half-lives (Figure 1A). These results indicate that both PE and PLA inhibited boscalid degradation in soil. To explore the mechanism, we conducted adsorption experiments and fitted the results using the Henry, Freundlich, and Langmuir models (Figure 1B,C, Table S6). For the boscalid–PE mixture, the Henry model exhibited a higher correlation coefficient compared to the Freundlich and Langmuir models. Analysis of the distribution coefficients (Kd) derived from the Henry model indicated that PE significantly increased boscalid adsorption affinity in soil. This conclusion was further supported by the other models. For the boscalid–PLA mixture, all three models provided reasonable fits, with the Freundlich model showing the highest correlation coefficient. This indicates that the BOS–PLA mixture in soil follows a heterogeneous, multilayer adsorption mechanism. With increasing PLA concentrations, the Freundlich exponent (*n*) varied, suggesting that at lower microplastic concentrations, multilayer adsorption is primarily driven by chemisorption, whereas at higher concentrations, adsorption becomes more favorable and is likely dominated by physisorption. Additionally, the Freundlich adsorption coefficient (KF) increased with rising PLA concentrations, providing additional evidence that PLA significantly enhanced the adsorption affinity of boscalid in soil. This conclusion is also supported by the fitting results obtained from the other models.

### 2.2. The Effect of Combined Exposure of Boscalid and/or PE/PLA on the Bioaccumulation of Boscalid

The degradation dynamics of boscalid were investigated in the presence of earthworms. Soil boscalid concentrations decreased rapidly from day 0–3, followed by a slower rate of decline after day 3. When PE was present, boscalid showed a higher half-life (69.31 days) compared with BOS alone (43.32 days), indicating a slower dissipation. When PLA was present, the half-life was 53.32 days, and it is higher than that in BOS alone. These results suggest that microplastics prolonged boscalid persistence in soil, while earthworms influenced its apparent dissipation (Figure 1D). Boscalid accumulation in earthworms was further analyzed. In the BOS and BOS + PE groups, boscalid concentrations initially declined from day 0–5, then gradually increased to a peak at day 21 before declining again (Figure 1E). In contrast, in the BOS + PLA group, boscalid concentrations increased from day 0–14, reaching a maximum, and then gradually declined. To assess the ability of earthworms to accumulate boscalid from soil, the Biota-Soil Accumulation Factor (BSAF) model was used. In the BOS group, the BSAF value increased from day 0–21 and then decreased from day 21–28. Under co-exposure with PE, the BSAF value decreased during days 1–3, increased to a peak at day 21, and then declined. Under co-exposure with PLA, the BSAF value increased until day 14 and then declined. Except for day 14, when the BSAF value in the co-treatment group was significantly higher than that in the BOS group, the BSAF values on other days were significantly lower. The BSAF values in both co-treatment groups were significantly higher than those in the BOS group (Table S7). In addition, the area under the concentration–time curve (AUC) was used to evaluate the bioavailability of boscalid. The AUC values for BOS, BOS + PE, and BOS + PLA were 15.53, 21.48, and 15.98 mg·d/kg, respectively. These findings indicate that PE significantly enhanced boscalid accumulation in earthworms. PLA significantly increased boscalid accumulation on day 14 but significantly decreased accumulation on other days. Overall, PLA did not significantly affect boscalid bioavailability in earthworms.

### 2.3. The Effect of Combined Exposure of Boscalid and/or PE/PLA on Intestinal Tissue

**Figure 2:**
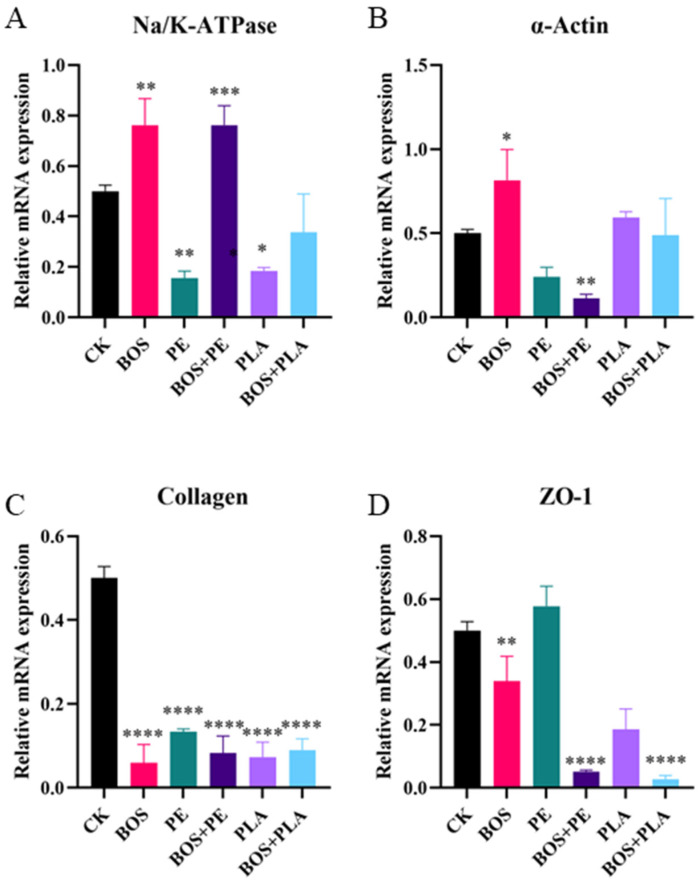
Effects of BOS and/or PE/PLA exposure on the expression of intestinal barrier-related genes in earthworms. Relative mRNA expression levels of *Na/K-ATPase* (**A**), *α-actin* (**B**), *Collagen* (**C**), and *ZO-1* (**D**) in earthworms after exposure to BOS, PE, PLA, or their combined treatments. Data are presented as mean ± SD (*n* = 6 independent biological samples collected from six independent beakers) * *p* < 0.05, ** *p* < 0.01, *** *p* < 0.001, **** *p* < 0.0001.

Next, the expression levels of four intestinal mRNAs in earthworms were measured. BOS exposure significantly upregulated the expression of *Na^+^/K^+^-ATPase* and *α-actin*, while significantly downregulating *Collagen* and *ZO-1* expression (Figure 2). Exposure to PE significantly downregulated *Na^+^/K^+^-ATPase*, *α-actin*, and *Collagen*. Co-exposure to BOS and PE resulted in significant upregulation of *Na^+^/K^+^-ATPase* and downregulation of *Collagen*, *ZO-1*, and *α-actin*. Exposure to PLA led to significant downregulation of *Na^+^/K^+^-ATPase*, *Collagen*, and *ZO-1*. In the BOS + PLA group, *Na^+^/K^+^-ATPase* expression was downregulated, *α-actin* was upregulated, and both *Collagen* and *ZO-1* were significantly downregulated. These results suggest that BOS may disrupt the intestinal barrier in earthworms. PE showed limited effects on these genes and did not markedly alter the response pattern observed under BOS exposure. In contrast, PLA showed a different expression pattern compared with BOS, and the combined exposure appeared to have a more pronounced effect on barrier-related gene expression. However, these interpretations are based on mRNA expression changes, and histological analysis of the earthworm gut would be needed to further validate the potential effects on intestinal barrier structure.

### 2.4. The Effect of Combined Exposure of Boscalid and/or PE/PLA on the Oxidative Stress

**Figure 3:**
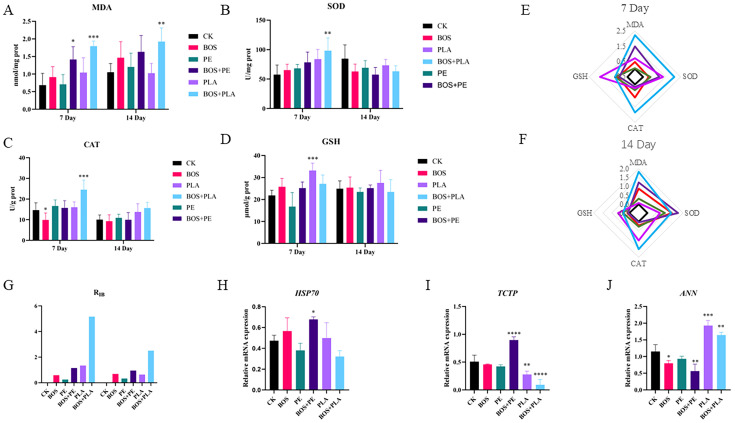
Effects of BOS and PE/PLA exposure on oxidative stress in the earthworm and the integrated biomarker response analysis. (**A**) Malondialdehyde (MDA) content. (**B**) Superoxide dismutase (SOD) activity. (**C**) Catalase (CAT) activity. (**D**) Glutathione (GSH) level. (**E**,**F**) Radar plots of integrated biomarker response (IBR) analysis on days 7 and 14, respectively. (**G**) IBR index values (RIB) in earthworms. (**H**–**J**) Relative mRNA expression levels of *HSP70*, *TCTP*, *ANN*. Data are presented as mean ± SD (*n* = 6 independent biological samples collected from six independent beakers per treatment) * *p* < 0.05, ** *p* < 0.01, *** *p* < 0.001, **** *p* < 0.0001.

Oxidative stress in earthworms was evaluated using biomarkers related to antioxidant defense and oxidative damage. Compared with the CK group, treatments with BOS, PE, BOS + PE, PLA, and BOS + PLA significantly increased MDA levels on both day 7 and day 14 (Figure 3A). CAT activity was elevated in the PE and BOS + PE groups, and significantly increased in the PLA and BOS + PLA groups on day 7, while CAT activity decreased in the BOS group. In addition, SOD activity increased on day 7 across all treatment groups (BOS, PE, BOS + PE, PLA, BOS + PLA), but declined by day 14. GSH levels increased in the BOS and BOS + PE groups on day 7, decreased in the PE group, and significantly increased in the PLA and BOS + PLA groups. By day 14, GSH levels across all treatment groups were similar (Figure 3B–D). Integrated biomarker response (IBR) analysis further showed that oxidative stress levels in the BOS + PLA group exhibited the highest oxidative stress levels among all treatment groups on both day 7 and day 14 (Figure 3E–G). Furthermore, the expression of three functional genes in earthworms was examined on day 14. *HSP70* and *TCTP* expression levels were upregulated in the BOS, PE, BOS + PE, and PLA groups but downregulated in the BOS + PLA group. Among them, the BOS + PE group showed the most pronounced upregulation. Regarding *ANN*, a reproduction-related gene, its expression was significantly downregulated following BOS and BOS + PE exposure, while significantly upregulated in the PLA and BOS + PLA groups (Figure 3H–J). These findings suggest that co-exposure with PE significantly enhanced BOS-induced oxidative stress. PE alone had limited effects on oxidative stress, and their role may primarily involve enhancing BOS bioaccumulation in earthworms through adsorption, thereby intensifying oxidative stress. In contrast, co-exposure with PLA not only increased BOS bioaccumulation but also directly contributed to oxidative stress, indicating a dual mechanism behind the observed intensification of BOS-induced oxidative damage.

### 2.5. The Effect of Combined Exposure of Boscalid and/or PE/PLA on Metabolic Disturbances

**Figure 4:**
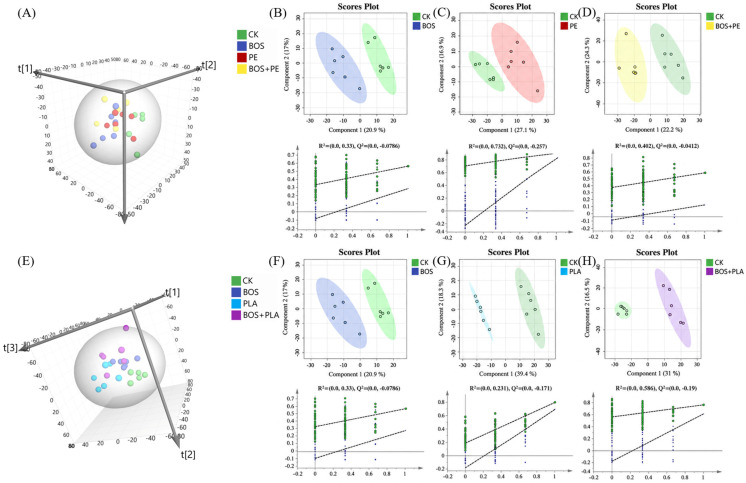
Effects of exposure to boscalid and/or microplastics on the metabolic profiles of earthworms. PCA score plots based on ^1^H NMR data are shown for the PE-related groups (CK, BOS, PE, and BOS + PE) (**A**) and PLA-related groups (CK, BOS, PLA, and BOS + PLA) (**E**). PLS-DA score plots with corresponding model validation plots are shown for CK vs. BOS (**B**,**F**), CK vs. PE (**C**), CK vs. BOS + PE (**D**), CK vs. PLA (**G**), and CK vs. BOS + PLA (**H**). Each point represents one biological replicate (*n* = 6 per treatment).

Untargeted metabolomics based on ^1^H NMR spectroscopy was conducted to evaluate metabolic changes in earthworms exposed to BOS and PE/PLA. The ^1^H-NMR spectra of earthworms are shown in Figure S1, with a total of 39 metabolites identified. Principal component analysis (PCA) score plots showed clear separation between the control and treatment groups, indicating that both single and combined exposures to BOS and PE/PLA significantly altered the metabolic profiles of earthworms and disrupted metabolic homeostasis (Figure 4).

**Figure 5:**
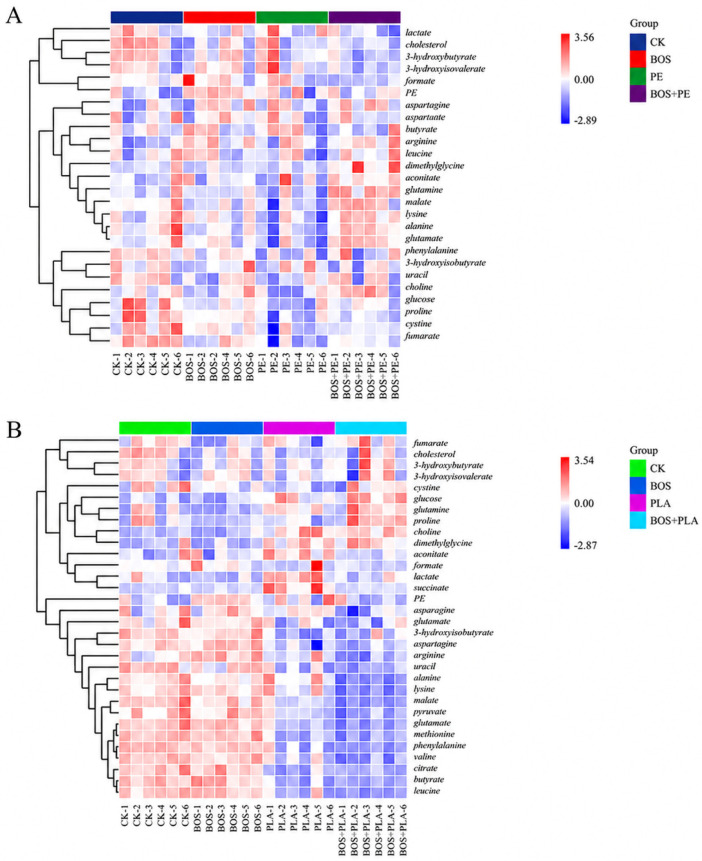
Effects of BOS and/or PE/PLA exposure on the metabolic profiles of earthworms. Heatmaps showing normalized relative abundances of significantly altered metabolites in earthworms exposed to BOS and/or PE (**A**) and BOS and/or PLA (**B**).

Pairwise PLS-DA comparisons were further conducted, and metabolites with variable importance in projection (VIP) values > 1 were selected as discriminant metabolites. PLS-DA model validation parameters and permutation testing results were provided in Table S8. In total, 26 and 32 significantly altered metabolites were identified, highlighting distinct metabolic profiles between the control and treatment groups. Specifically, in the BOS group, 10 metabolites were significantly altered, including 7 downregulated (uracil, tyrosine, phenylalanine, lysine, glycogen, fumarate, cysteine) and 3 upregulated (phosphatidylethanolamine, leucine, arginine). In the PE group, 10 metabolites were also significantly altered, with 7 downregulated (cholesterol, leucine, lysine, alanine, glutamate, aspartate, fumarate) and 3 upregulated (butyrate, 3-hydroxybutyrate, 3-hydroxyisovalerate). In the BOS + PE group, 20 metabolites were significantly altered, including 10 downregulated (cholesterol, 3-hydroxyisobutyrate, 3-hydroxyisovalerate, lactate, aspartate, phenylalanine, glycogen, fumarate, tyrosine, formate) and 10 upregulated (leucine, lysine, arginine, glutamate, glutamine, malate, dimethylglycine, asparagine, cis-aconitate, choline). For the PLA group, 27 metabolites were significantly altered, with 23 downregulated (cholesterol, butyrate, leucine, valine, arginine, glutamate, glutamine, methionine, maleate, pyruvate, 3-hydroxyisovalerate, 3-hydroxyisobutyrate, citrate, aspartate, asparagine, lysine, phenylalanine, glucose, glycogen, cysteine, uracil, fumarate, tyrosine) and 4 upregulated (succinate, lactate, choline, dimethylglycine). In the BOS + PLA group, 28 metabolites were significantly altered, including 20 downregulated (butyrate, leucine, valine, 3-hydroxyisobutyrate, lactate, lysine, arginine, glutamate, glutamine, methionine, maleate, pyruvate, citrate, aspartate, asparagine, cysteine, phenylalanine, alanine, uracil, formate) and 8 upregulated (3-hydroxyisovalerate, succinate, dimethylglycine, tyrosine, phosphatidylethanolamine, glucose, glycogen, choline) (Figure 5).

In addition, metabolic pathway enrichment analysis was conducted on the 26 significantly altered metabolites (Figures S2 and S3). Pathway enrichment analysis was performed with statistical correction for multiple testing to reduce false-positive results. BOS exposure resulted in the significant enrichment of five metabolic pathways: phenylalanine, tyrosine, and tryptophan biosynthesis; the tricarboxylic acid (TCA) cycle; phenylalanine metabolism; pantothenate and coenzyme A biosynthesis; and glyoxylate and dicarboxylate metabolism. PE exposure was associated only with the enrichment of butyrate metabolism. Combined exposure to BOS + PE significantly enriched six pathways: alanine, aspartate, and glutamate metabolism; glyoxylate and dicarboxylate metabolism; arginine biosynthesis; phenylalanine, tyrosine, and tryptophan biosynthesis; the TCA cycle; and phenylalanine metabolism. PLA exposure significantly enriched six pathways: alanine, aspartate, and glutamate metabolism; glycine, serine, and threonine metabolism; arginine biosynthesis; phenylalanine, tyrosine, and tryptophan biosynthesis; phenylalanine metabolism; and glyoxylate and dicarboxylate metabolism. BOS + PLA exposure significantly enriched five pathways: glyoxylate and dicarboxylate metabolism; alanine, aspartate, and glutamate metabolism; the TCA cycle; pantothenate and coenzyme A biosynthesis; and glycine, serine, and threonine metabolism.

**Figure 6:**
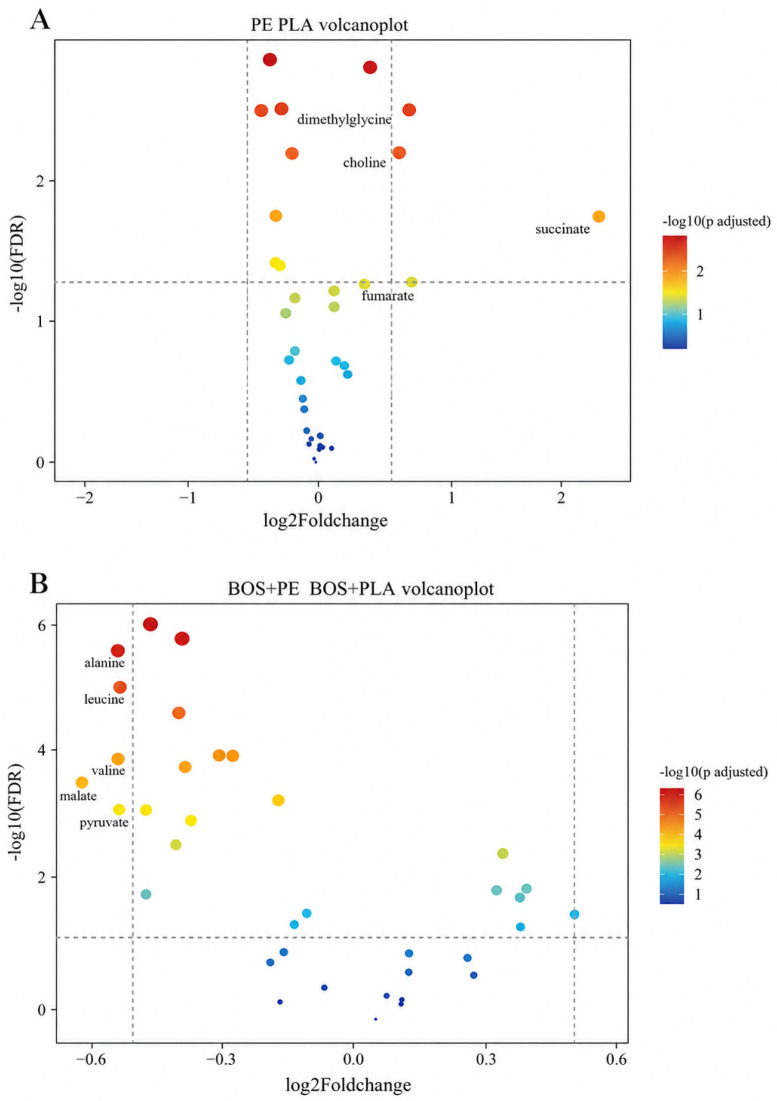
Differential metabolite analysis in earthworms exposed to PE/PLA and BOS + PE/BOS + PLA. (**A**) Volcano plot showing significantly altered metabolites between the PE and PLA groups. (**B**) Volcano plot showing significantly altered metabolites between the BOS + PE and BOS + PLA groups.

Subsequently, PLS-DA models based on metabolites from the PE, BOS + PE, PLA, and BOS + PLA groups were constructed. The results showed clear separations between PE and PLA, as well as between BOS + PE and BOS + PLA. Volcano plots were then generated to identify differential metabolites caused by PLA and PE in the presence of boscalid (Figure 6). Compared with the PE group, the PLA group exhibited significant increases in dimethylglycine, choline, fumarate, and succinate. Compared with the BOS + PE group, the BOS + PLA group showed significant decreases in alanine, valine, leucine, maleate, and pyruvate. These results indicate that co-exposure to BOS and MPs induced more pronounced metabolic disturbances in earthworms. The metabolic disruptions caused by PE and PLA are not solely attributable to the enhanced pesticide bioavailability due to adsorption but also involve distinct metabolic perturbations differing from single exposures. Moreover, PLA and PE exert different mechanisms of action on earthworm metabolism.

## 3. Discussion

Microplastics have received great attention in agricultural ecosystems due to their widespread presence and ecological impacts [25]. They can adsorb and accumulate various organic and inorganic compounds [26], which may increase the ecotoxicological risks of these pollutants. Biodegradable plastics have been developed as alternative materials to solve this problem [27]. Therefore, we attempt to explore whether biodegradable microplastics can truly solve the hazards of traditional plastic products. In this study, PE and PLA were selected to evaluate how conventional and biodegradable microplastics influence the environmental behavior of BOS in soil. According to previous research, BOS had low adsorption in soil, and because the soil contains more metal ions, it has a higher adsorption rate for negatively charged PE [28]. As a result, PE addition significantly increased BOS adsorption in soil. At the same time, the adsorption of PE and BOS in soil was more consistent with the Henry model among the three models, indicating that the adsorption of PE and BOS complex by soil was approximately linear, which may be mainly due to hydrophobic partitioning and van der Waals forces [29]. Compared with PE, PLA was better fitted by the Freundlich model, indicating a heterogeneous distribution of adsorption sites, which may be related to the heterogeneity of the surface biofilm [30]. The adsorption coefficient of PLA in each model was basically higher than that of PE, indicating that PLA could significantly increase the adsorption of BOS in soil. This may be attributed to the negative charge of the carboxyl group of PLA, which is easier to bind to soil sites [31]. In addition, the functional group of PLA can form ionic bonds or hydrogen bonds with BOS to adsorb it to a greater extent [32,33]. However, these explanations should be regarded as possible mechanisms, because isotherm fitting alone cannot determine the dominant adsorption forces. Further physicochemical characterization, such as FTIR, XPS, or SEM analysis, would be needed to clarify the detailed adsorption mechanisms.

BOS degradation in soil was further investigated in the influence of PE and PLA. Similar to the previous report by Ju et al. [34], both PE and PLA significantly prolonged the degradation half-life of BOS and inhibited its dissipation in soil [34]. This may be explained by the adsorption of BOS onto the surface of PE, which reduces its bioavailability to soil microorganisms [35]. Compared with PE, PLA caused a longer half-life of BOS, possibly due to its higher BOS adsorption and potential influence on microbial community structure and enzyme activity [36]. Previous studies have shown that soil microorganisms are important contributors to the biodegradation of organic pollutants [37]. However, soil microbial communities and enzyme activities were not directly measured in this study and further validation through microbiome and soil enzyme analyses were needed. In earthworm-containing systems, the reduction in boscalid residues in soil should be interpreted as apparent dissipation rather than direct degradation, as it may result from multiple coupled processes including microbial transformation, adsorption to soil and microplastics, uptake by earthworms, and redistribution between soil and organisms. Therefore, in soil-only systems, both PE and PLA inhibited BOS degradation, and PLA exhibited a stronger inhibitory effect than PE, indicating a higher potential ecotoxicological risk.

We further explored the interaction between soil organisms and environmental pollutants. Previous studies have shown that earthworms can transport and ingest plastic fragments in soil [38], and earthworm ingestion can promote further fragmentation of microplastics [39]. Therefore, we investigated how earthworms influenced BOS degradation in the presence of PE and PLA. The results showed that earthworms can effectively promote BOS degradation in soil. Compared with PE, PLA led to a more significantly decrease in the half-life of boscalid in the BOS + PLA group, which may be due to the preference of earthworms for PLA monomers or polymers under starvation conditions [40]. Unlike PE, the enrichment of boscalid by earthworms in the BOS + PLA group was initially lower than that in the BOS group, then increased before declining again, and the overall enrichment capacity did not differ significantly from the BOS group. The BSAF results suggested that the BOS and BOS + PE groups showed an initial decrease followed by a subsequent increase, indicating that the ability of earthworms to metabolize pesticides may be weakened in the early stage of exposure, resulting in increased bioaccumulation. To further assess the adverse effects of BOS on earthworms, bioavailability was evaluated, as it is considered an important parameter for estimating the risks of pesticide residues ingestion [41]. The results showed that microplastics can act as carriers to adsorb pesticides and invade the bodies of earthworms through feeding behavior, thereby increasing the bioavailability of BOS to earthworms and increasing ecological risks. In the BOS + PLA group, BSAF and bioavailability were significantly higher than those of the BOS group on day 14, but were significantly lower at most of the remaining days. This pattern may be partly explained by the avoidance behavior of earthworms toward PLA plastics [42]. In the first few days, the degree of hunger stress is low, so earthworms try to avoid ingesting PLA. As exposure time increases, hunger stress becomes stronger and PLA produces free monomer lactic acid, which may increase the attractiveness of PLA to earthworms and promote PLA ingestion. In addition, earthworms are more likely to decompose bio-based microplastics than fossil-based microplastics [40]. Compared with the exposure time, the half-life of earthworms excreting PLA is shorter, so the utilization of BOS decreases again. Overall, this study believes that during certain exposure periods, biodegradable microplastics may cause more serious ecological risks than traditional microplastics.

Previous studies have shown that ingestion or accumulation of microplastics in soil animals can cause physical tearing of organs and tissues and trigger an inflammatory response [43]. Therefore, we assessed the expression of intestinal functional genes in earthworms. Similar to previous findings [44], microplastics exposure significantly inhibited the expression of *Na/K-ATPase*. Similar expression levels were observed in the BOS and BOS + PE groups. According to above results, BOS exposure upregulated *Na/K-ATPase* expression, indicating that for environmental concentrations of BOS and PE, the dominant effect of toxicity may come from BOS. In contrast, the toxicity of the BOS + PLA group is closer to that of the PLA group, indicating that its dominant effect may come from PLA. Moreover, for earthworm intestinal protein genes, BOS + PE exposure significantly inhibited the expression of *ZO-1*, *α-actin* and *Collagen*. Insufficient *ZO-1* protein may lead to intestinal permeability and dysfunction because it recruits other tight junction proteins [45]; down-regulation of *Collagen* expression may lead to decreased integrity of the earthworm intestinal barrier; and down-regulation of *α-actin* may lead to weakened intestinal peristalsis. Compared with PE, PLA induced a stronger decrease in *ZO-1* expression, and BOS + PLA co-exposure further reduced *ZO-1* and *Collagen* levels, which is consistent with the previous report that exposure to PLA causes atrophy of the earthworm circular muscles [46]. In general, damage to the intestinal barrier may lead to accelerated invasion and accumulation of exogenous pollutants in the body [47]. On the one hand, PE and PLA may cause damage to the intestinal cavity of earthworms. On the other hand, microplastics, as carriers for adsorbing pollutants [48], promote the enrichment of BOS in earthworms, improve the bioavailability of BOS, and increase ecological risks.

Environmental pollutants can cause not only tissue damage but also excessive free radical production in cells, which is one of the main factors causing oxidative stress [49], and the number of free radicals is correlated with the concentration of environmental pollutants [50]. We further measured the antioxidant system function of earthworms and the content of MDA, a marker of oxidative stress. The results showed that the MDA levels in the combined exposure group were higher than those in the single-exposure group during the 0–14 day exposure period, which is consistent with the previous study reporting that MDA levels increase within 0–14 days under combined exposure to microplastics and pesticides [51]. Antioxidant enzyme activities in earthworms were further evaluated. SOD is generally considered to be a key enzyme in resisting superoxide radicals caused by external pollutants [52]. In this study, SOD activity increased on day 7 but inhibited by day 14 in the exposure groups. The reason may be that the accumulated reactive oxygen species inhibited the function of SOD and led to an increase in MDA levels [53]. CAT can decompose H_2_O_2_ into H_2_O and O_2_ and works together with SOD as part of the first-line antioxidant defense system [54]. On day 7, CAT activity decreased significantly in the BOS group but increased markedly in the BOS + PLA group. Previous studies have reported that microplastic exposure can promote the increase in CAT activity, and this effect can still be exerted in the presence of other pollutants, which is similar to the results of this study [55]. GSH is widely used in animals to detoxify ROS, exogenous biotin, and reactive intermediates of cellular metabolism [56]. PLA promoted an increase in GSH levels, possibly through effects on the TCA cycle and antioxidant defense. The expression of stress resistance- and reproduction-related genes was then analyzed, and the results showed that *HSP70* expression increased. It has been shown that upregulation of *HSP70* protein expression can induce ferroptosis, which may be one of the causes of oxidative stress [57]. In addition, transcriptionally controlled tumor protein (*TCTP*) is a highly conserved protein expressed in eukaryotes. The increased expression of *TCTP* indicated a disrupted regulatory response caused by the combined exposure [58]. *ANN* encodes an egg-laying hormone-like peptide and serves as an important reproductive biomarker in earthworms [59]. Similar to previous reports, *ANN* downregulation suggests a survival strategy of earthworms to reduce reproductive behavior and conserve energy under high environmental stress [60]. These findings suggest that co-exposure to boscalid and PLA may be associated with increased stress responses in earthworms and enhanced antioxidant activity, indicating a potential increase in oxidative stress.

Oxidative stress is generally associated with changes in endogenous metabolites [61]. In this study, BOS exposure decreased the accumulation of fumaric acid and maleic acid in earthworms and caused a slight increase in the level of aconitic acid. PE exposure caused a more significant decrease in fumaric acid and maleic acid. This effect may be associated with the effect of PE on citrate synthase and pyruvate carboxylase [62], or with the indirect effect of glutamate and glutamine decomposition caused by PE on the TCA cycle [39]. The significant decrease in glutamate, glutamine, and maleic acid levels in the PE group support this conclusion. Therefore, the inhibitory effect of BOS and PE on different sites of the TCA cycle may be the reason why the trends of TCA cycle metabolites in the combined treatment group are different from those in the single treatment group. This could explain why fumaric acid level in the combined treatment group is between the single treatment groups. We then focused on observing metabolites with different trends from the single exposure group to explore the unique effects of combined exposure. First, changes in glutamate, glutamine, and malate suggest that combined exposure may cause more severe oxidative stress in earthworms, consistent with previous findings. It has been reported that increased glutamate levels may be one of the reasons for inducing oxidative stress [1]. Earthworms try to cope with higher metabolic pressure by inducing glutamate to convert into glutamine and consuming cysteine to synthesize GSH [63]. The increase in glutamate levels leads to the replenishment of malate levels, which may be one of the strategies for earthworms to maintain energy and redox balance. Dimethylglycine is one of the important substances for maintaining osmotic balance and can also serve as a glycine source for the synthesis of detoxification-related molecules, such as glutathione [64]. For the combined treatment group, the increase in dimethylglycine not only compensates for the osmotic pressure imbalance caused by abnormal *Na/K-ATPase* expression, but also participates in the synthesis of reduced glutathione as a glycine source to cope with environmental pressure. Alanine is an important metabolic intermediate that participates in biochemical reactions such as gluconeogenesis and amino acid metabolism [65]. Combined treatment may aggravate the energy crisis, activate the MAPK pathway, promote glycolysis, lead to pyruvate accumulation, and then generate more alanine through the transamination reaction catalyzed by ALT [66]; another pathway may come from the consumption of excess glutamate by earthworms. Alanine also acts as an important osmotic protectant [67], and its increased level can help maintain osmotic balance under environmental stress. Therefore, combined exposure to BOS and PE may induce more severe metabolic disorders in earthworms than single exposure. This may be because BOS and PE affect different biological targets, resulting in more complex toxic effects and increased ecological risk.

In addition, PLA significantly decreased the levels of malic acid and citric acid, while increasing succinic acid levels. This may suggest that the lactic acid produced by the decomposition of PLA by earthworms enters the TCA cycle through glycolysis to provide partial energy compensation [68], which corresponds to the significant increase in the level of lactic acid noted in this study. Regarding the TCA cycle, malic acid in the combined exposure group decreased most significantly, whereas fumaric acid levels were partially recovered. Citric acid levels was similar to that of the PLA group, and the levels of other metabolites were similar to those of the BOS group, indicating that the combined action of BOS and PLA may inhibit the biochemical reaction process from fumaric acid to malic acid. Oxidative phosphorylation, glucose metabolism, and tricarboxylic acid metabolism work together to form the energy metabolism chain in cells [69]. This study noted the increase in glucose and glycogen levels and the decrease in pyruvic acid levels in the PLA and BOS + PLA groups, suggesting that PLA may affect the oxidative phosphorylation process, which may have caused a more serious energy gap for earthworms. Meanwhile, most amino acids in the PLA group and BOS + PLA groups decreased significantly, which suggest that energy deficiency may forces earthworms to activate the gluconeogenesis pathway to meet the energy needs of survival [70]. This may be one of the survival strategies of earthworms in the face of energy deficiency. After that, this study further constructed differential metabolic volcano maps of the PE, PLA, BOS + PE, and BOS + PLA groups to explore the unique effects of different microplastics. Compared with the PE group, the PLA group found a significant increase in fumaric acid, succinic acid, dimethylglycine, and choline. It is reported that polyethylene microplastics increase choline metabolism, while polylactic acid microplastics can prevent this process [71]. Dimethylglycine is one of the important substances for maintaining osmotic balance and one of the metabolites affected by intestinal microorganisms. It is reported that PLA exposure can significantly increase the α diversity of intestinal flora compared with PE [72]. The significant increase in dimethylglycine may be related to intestinal barrier disruption and changes in the abundance of specific bacterial genera. Compared with the BOS + PE group, alanine, valine, leucine, maleic acid, and pyruvic acid were significantly reduced in the BOS + PLA group. This result suggests that under short-term exposure, the decomposition of PLA cannot provide enough lactic acid to supplement the energy deficiency of earthworms but instead causes a more serious energy deficit. Therefore, compared with PE, the combination of PLA and BOS caused more serious metabolic disorders.

However, our study has several limitations that should be considered. First, the soil–earthworm experiment was conducted as a controlled artificial soil microcosm using one boscalid dose and one PE/PLA dose over a 28-day exposure period. Therefore, the results mainly reflect the responses observed under the tested laboratory conditions and cannot be directly extrapolated to dose-dependent effects, long-term exposure, or field conditions. Second, the biological endpoints measured in this study mainly included intestinal barrier-associated gene expression, oxidative stress biomarkers, and metabolomic profiles, whereas organism-level endpoints such as survival, growth, reproduction, avoidance behavior, and feeding activity were not examined. Future studies should include multiple exposure concentrations, natural agricultural soils, longer exposure periods, LC-MS/MS-based metabolomics and organism-level endpoints to more comprehensively evaluate the ecological risks of boscalid and microplastic co-exposure.

## 4. Materials and Methods

### 4.1. Reagents and Chemicals

Boscalid (BOS, purity ≥ 98%) was supplied by Tianjin Xiens Technology Co., Ltd. (Tianjin, China) Polyethylene microplastics (PE, CAS: 9002-88-4) and Polylactic acid microplastics (PLA, CAS: 26100-51-6) were purchased from Sigma-Aldrich (Merck, Boston, MA, USA). The physicochemical characteristics of PE and PLA microplastics, including particle size information, surface morphology, specific surface area, zeta potential, and aging status, are summarized in Table S1. The microplastics used in this study were pristine commercial particles and were not artificially aged before exposure. *Eisenia fetida* earthworms were acquired from Dongli Biotechnology Co., Ltd. (Tianjin, China).

### 4.2. Experiment Design

Experiment 1: The experiment consisted of a blank control, a BOS treatment (4 mg/kg dry weight), PE and PLA treatments (0.1% dw), and combined treatments of BOS with PE or PLA (4 mg/kg dw BOS + 0.1% dw PE/PLA). Each experimental group was prepared either without earthworms or with ten *Eisenia fetida*. Detailed microcosm setup is provided in Table S3. Each replicate contained 300 g of prepared artificial soil in a 500 mL beaker, with deionized water added to maintain 35% soil moisture. Three independent beakers were prepared for each treatment at each sampling time. Sampling was destructive; therefore, separate beakers were used for different sampling days. Soil and organism samples were collected on days 1, 3, 5, 7, 14, 21, and 28.

Experiment 2: Boscalid adsorption experiments were conducted in accordance with the OECD 106 method. 2.0 g of dry soil was mixed with 10 mL of boscalid standard solution prepared in 0.01 M CaCl_2_ as the background electrolyte. The initial boscalid concentrations were set at 0.5, 1, 2, 4, and 8 mg/L. To evaluate the influence of microplastics on boscalid adsorption, separate treatments containing PE or PLA at 0.1%, 0.5%, and 1% dw were prepared. All tubes were continuously agitated at 25 ± 1 °C for 24 h, and centrifuged at 8000 rpm for 5 min. 1.0 mL aliquot of the supernatant was filtered through a 0.22 μm membrane before analysis by high-performance liquid chromatography (HPLC). Each treatment was conducted in triplicate [73].

Blank and control treatments were also included to evaluate possible background interference and non-soil losses of boscalid. The soil blank contained soil and 0.01 M CaCl_2_ without boscalid or microplastics. The boscalid solution control contained boscalid in 0.01 M CaCl_2_ without soil or microplastics and was used to assess possible losses caused by tube-wall adsorption or sample handling. In addition, boscalid + PE and boscalid + PLA controls without soil were prepared to evaluate boscalid adsorption by microplastics alone. All blank and control treatments were processed under the same shaking, centrifugation, filtration, and analytical conditions as the adsorption treatments.

Experiment 3: This experiment followed the same design as Experiment 1. For earthworm sample collection, individuals were removed from the beakers and placed on moist filter paper for 3 h to clear their gut contents. Then, earthworms were collected and allowed to depurate on moist filter paper for 3 h. The depurated earthworms were then gently blotted, snap-frozen in liquid nitrogen, and stored at −80 °C. Soil samples from the corresponding beakers were collected and preserved under the same conditions. For qPCR, oxidative stress biomarker, and metabolomics analyses, six independent beakers were prepared for each treatment at the corresponding sampling time. One biological sample was collected from each independent beaker; *n* = 6 biological replicates.

The concentrations of boscalid and microplastics used in this study were selected based on previously reported microcosm and soil exposure experiments [74]. The boscalid concentration was chosen to ensure detectable levels during the experimental period and to allow reliable kinetic analysis of its dissipation behavior. Although the microplastic concentration used in this study (0.1%) is higher than typical environmental levels, it is consistent with levels commonly used in controlled soil microcosm experiments to ensure measurable biological and chemical responses under laboratory conditions [75,76]. Exposure soils were prepared by spiking quartz sand with methanol solutions containing boscalid, microplastics, or their mixtures. After the methanol had evaporated completely in a fume hood, the spiked quartz sand was homogenized with the other soil constituents. Artificial soil was prepared following OECD (207) guidelines, comprising 10% well-sieved algae soil, 20% kaolin and 70% 0.05–0.2 mm quartz sand.

### 4.3. Sample Extraction and Analysis

#### 4.3.1. Boscalid Extraction

We used a modified QuEChERS method to extract boscalid, and the detailed protocol is provided in Supporting Information S1.

#### 4.3.2. Instrumental Conditions

Boscalid was quantified by a UHPLC-UltiMate 3000 and a TSQ Quantum Access MAX system (Thermo Fisher, Waltham, MA, USA). Detailed analytical conditions are provided in Supporting Information S2.

We developed a method to analyze the fate of boscalid in soil and earthworms, with the validation results summarized in Tables S3 and S4.

#### 4.3.3. Quantitative Real-Time PCR (qRT-PCR) Analysis

Seven functional genes were measured using qRT-PCR analysis. *β-Actin* was used as the housekeeping gene, and relative gene expression levels were calculated using the 2^−ΔΔCt^ method. A list of primers and detailed information on the qRT-PCR analysis are provided in Table S5. Total RNA was extracted and reverse-transcribed using TIANGEN kits. qRT-PCR was performed on a Bio-Rad (Hercules, CA, USA) CFX 96 PCR system.

### 4.4. Analysis of Oxidative Stress Parameters

Total protein content, SOD activity, CAT activity, GSH levels, and MDA concentrations were measured following the kit protocols. Six biological replicates were analyzed for each treatment group.

### 4.5. Metabolomics Analysis

We performed untargeted metabolomics using ^1^H NMR spectroscopy. Samples were prepared according to the procedures described in Supporting Information S3 and S4. One-dimensional spectral data were processed using Mestrenova software (version 6.1.0-6224). Multivariate statistical analyses were conducted with SIMCA-P 13.0 (Umetrics, Malmö, Sweden) and MetaboAnalyst 6.0. Metabolite identification confidence was ensured by matching chemical shifts with published literature and reference databases for ^1^H NMR spectra.

### 4.6. Statistical Analysis

Data are expressed as mean ± SD. Boscalid degradation was modeled using a first-order kinetic equation (C = C_0_ × e^−kt^, C: concentration, C_0_: initial concentration, k: digestion coefficient, t: digestion time), the half-life T = ln2/k. Normality and homogeneity of variance were assessed by the Shapiro–Wilk and Levene’s tests. Group comparisons utilized one-way ANOVA followed by Tukey’s post hoc test using SPSS 25.0 (IBM, Armonk, NY, USA). When the data did not meet the assumptions for parametric analysis, the non-parametric Kruskal–Wallis test followed by Dunn’s post hoc test was used. *p* value < 0.05 is considered statistically significant.

## 5. Conclusions

Overall, co-exposure to boscalid and microplastics enhanced boscalid adsorption and slowed its dissipation in soil, which enhances boscalid accumulation in earthworms. This process was accompanied by intestinal barrier impairment, oxidative stress, and metabolic disorders. Compared with PE, PLA showed more pronounced effects in certain endpoints. These results indicate that interactions between boscalid and microplastics may amplify their ecological risks in soil environments, highlighting the need for further studies to clarify their specific mechanisms of action.

## Data Availability

The raw data supporting the conclusions of this article will be made available by the authors on request.

## Supplementary Materials

The following supporting information can be downloaded at https://www.mdpi.com/article/10.3390/molecules31132268/s1. Ref. [77] is cited in the Supplementary Materials.
