# Supplementary material for: Comparative Impacts of Conventional and Biodegradable Microplastics on Boscalid Behavior and Toxicity in Soil–Earthworm System
Source: Molecules. 2026 Jun 29;31(13):2268. doi: 10.3390/molecules31132268 (PMC13363563; doi:10.3390/molecules31132268)
Supplement: Supplementary file 1 [file molecules-31-02268-s001.zip › molecules-4342092-supplementary.pdf]

Table of contents

Supporting information S1. Extraction of BOS from soil samples and earthworm samples.

Supporting information S2. Instrument condition.

Supporting information S3. The ^{1}H NMR Metabolomic samples preparation method.

Supporting information S4. The instrument parameters of Burkev AV III 600 NMR and processing methods ^{1}H NMR spectrums.

Table S1. Physicochemical characteristics of PE and PLA microplastics.

Table S2. Experimental Design for the Microcosm.

Table S3. Linearity of boscalid in earthworm.

Table S4. Spiking recoveries of boscalid in earthworm.

Table S5. Primer information.

Table S6. Effect of microplastics on the adsorption behaviors of boscalid (BOS) in experimental soil.

Table S7. Bio-soil accumulation factor (BSAF) of boscalid in earthworms.

Table S8. PLS-DA model validation parameters and permutation testing results.

Figure S1. Representative 600MHz ^{1}H NMR spectra of earthworm samples.

Figure S2. Enrichment of metabolic pathways of differential metabolites in earthworms.

Figure S3. Enrichment of metabolic pathways of differential metabolites in earthworms

Supporting information S1. Extraction of BOS from soil samples and earthworm samples.

Earthworm samples (0.4 g per sample) were freeze-dried, homogenized, centrifuged at 12,000 rpm for 10 min, and transferred to 15 mL centrifuge tubes. Then, add 2 mL acetonitrile, 0.4 g sodium chloride, and 0.4 g anhydrous magnesium sulfate. The mixture was vortexed for 10 minutes and centrifuged at 3800 rpm for 5 minutes. Transfer 2 mL of supernatant to a new centrifuge tube. The supernatant was frozen in a -20 °C refrigerator for 2 hours and centrifuged at 12,000 rpm for 10 minutes at 4°C. Finally, the supernatant was filtered through a 0.22μm membrane and subjected to LC-MS/MS analysis.

Extraction of boscalid from soil samples.

Soil samples (2 g per sample) were weighed and transferred to 15 mL centrifuge tubes. Then, add 4 ml acetonitrile, 1 g sodium chloride and 0.5 g anhydrous magnesium sulfate. Vortex the mixture for 10 minutes, sonicate it for 5 minutes, let it stand for 2-3 hours, and centrifuge it at 3800 rpm for 5 minutes. Add 1 ml of supernatant to a new centrifuge tube and 50 mg of PSA for purification. Finally, the sample solution was filtered through a 0.22-μm membrane and subjected to LC-MS/MS analysis.

Supporting information S2. Instrument condition.

Boscalid was separated in isocratic elution mode using a Waters ACQUITY UPLC BEH C18 Column (2.1 mm X 100 mm, 1.7 μm), and the mobile phase consists of acetonitrile/0.1% formic acid in water = 70/30 (v/v). The flow rate was 0.3 mL/min, and the injection volume was 10 μL. MS conditions were in selected reaction monitoring (SRM) scan mode: spray voltage of 3000 V for positive polarity, capillary temperature of 350 °C, vaporizer temperature of 50 °C, sheath gas pressure of 15 psi, auxiliary gas pressure of 15 psi, Q2 collision gas pressure is 1.5 mTorr. The mass spectrometric conditions were as follows: precursor ion at 343.0 (m/z), products 140.1 (m/z), and 271.2 (m/z) for quantification and characterization.

Supporting information S3. The ^{1}H NMR Metabolomic samples preparation method.

The earthworm tissue was lyophilized, homogenized and then extracted with 1.2 mL of phosphate buffer (5 mM, with TSP-d4). The buffer was prepared in 99% pure D2O from Cambridge Isotope Laboratories (Tewksbury, MA, USA) and then centrifuged twice at 21,000×g for 20 min. From each sample, 40 μL was withdrawn and pooled as a QC sample.

Supporting information S4. The instrument parameters of Burkev AV III 600 NMR and processing methods ^{1}H NMR spectrums.

The nuclear magnetic resonance spectroscopy was performed on a Bruker Avance III 600 NMR spectrometer (600 MHz, plus cryoprobe). All spectra were collected using a pre-saturated 1D Nuclear Overhauser Enhancement Spectroscopy (1D NOESY) pulse sequence to suppress the water peak. The acquired spectral width was 20 ppm. For each plot, 128 scans were acquired into 64k of data, with a relaxation time of 4s and a scan time of 2.66 s.

Table S1. Physicochemical characteristics of PE and PLA microplastics.

Property	PE	PLA	Source
Polymer type	Polyethylene	Polylactic acid	Supplier information
CAS No.	9002-88-4	26100-51-6	Supplier information
Particle size / distribution	34-50 μm	20-50 μm	Supplier information
Surface morphology	irregular fragments with relatively smooth surface	Spheroidal particles with slight surface undulations and slightly rough edges	Supplier information
Specific surface area	0.81 m^2/g	0.92 m^2/g	Supplier information
Zeta potential	-18.3 mV	-32.7 mV	Zeta potential analyzer
Aging status	Pristine, non-aged	Pristine, non-aged	Experimental design

Table S2. Experimental Design for the Microcosm.

Group	Abbreviation	Reagent	Number of earthworms (soil earthworm microcosms)	Number of earthworms (soil microcosms)
Control check	CK		10	0
Boscalid	BOS	4mg/kg boscalid	10	0
PE-MPs	PE	0.1%dw PE	10	0
PLA-MPs	PLA	0.1%dw PLA	10	0
Boscalid+PE-MPs	BOS+PE	4mg/kg boscalid+0.1%dw PE	10	0
Boscalid+PLA-MPs	BOS+PLA	4mg/kg boscalid+0.1%dw PLA	10	0

Note: concentrations were measured by soil dry weight

Table S3. Linearity of boscalid in earthworm.

Matrix	Linear equation	R^2	LOD（mg/kg）	LOQ（mg/kg）	Matrix effect（%）
Methanol	y = 16081904.23x + 984577.6667	0.9958	0.0001	0.005	
Soil	y = 13223515.93x + 815444.6207	0.9991	0.0001	0.005	-17.77
Earthworm	y = 9221474.121x - 137445.9751	0.9997	0.0001	0.005	-42.66

Table S4. Spiking recoveries of boscalid in earthworm.

Samples	Spiking levels (mg/kg)	Average recoveries (%)	Standard deviation (%)
Soil	4	92.26±8.80	9.54
	1	90.77±4.29	4.73
	0.25	85.48±3.78	4.42
Earthworm	0.5	104.14±5.22	5.02
	0.125	121.54±6.36	5.24
	0.0625	100.64±3.59	3.57

Table S5. Primer information.

Gene	GenBank accession no.	Gene-specific primers (5′–3′)	Efficiency	R^2	Specificity
ACTB	GU177854	F:GTTCGAAACCTTCAACTCCC<br>R:TGGTGGTGAAGCTGTAGCCT	96.5%	0.996	single melting peak
Hsp70	GU177858	F:GCCATGAACCCAACCAACAC<br>R:TGGATCTTCGGCTTGCCATT	98.2%	0.994	single melting peak
TCTP	GU177860	F:AGAGACCAAACCGGCAGACA<br>R:TCGCCACAGAAGAGCTGGTAT	102.1%	0.991	single melting peak
ZO-1	GU177859	F:TTTTCCGCATCTGTTCCTCCT<br>R:CTCGGTCCGTTGTTCCTTGAC	99.6%	0.994	single melting peak
Na^+/K^+-ATPase	Yang et al. (2020)	F:GAGACAGATGGCAAAGAACCC<br>R:AACGCAAAGACAAAGAAGAGC	99.4%	0.996	single melting peak
α-Actin	Yang et al. (2020)	F:GATAGCAAACCGCAAGATGAT<br>R:TGAAAGCAAGGGCGTAAAACT	98.6%	0.991	single melting peak
ANN	Yang et al. (2020)	F:TTTCTTCCGCCTGCTTTG<br>R:ACCGACCTACCACCGACA	98.3%	0.998	single melting peak
Collagen	Yang et al. (2020)	F:CACCAACAGGTCCTGTGACTC<br>R:TACTGGTCCACAAGGTTCTAAA	99.6%	0.997	single melting peak

Reference:

Yang, Y.; Liu, P.; Li, M., Tri-n-butyl phosphate induced earthworm intestinal damage by influencing nutrient absorption and energy homeostasis of intestinal epithelial cells. Journal of hazardous materials 2020, 398, 122850.

Table S6. Effect of microplastics on the adsorption behaviors of boscalid (BOS) in experimental soil.

MPs (dw)	Henry		Freundlich			Langmuir		
	K_d	R^2	K_f	n	R^2	K_L	Q_{max}	R^2
0	1.8322	0.9447	0.599653	0.640205	0.9774	0.3035	0.98834	0.9063
0.10% PE	2.1393	0.9704	0.892278	0.665735	0.8913	0.36057	0.9522	0.7684
0.50%PE	2.1509	0.9877	1.047611	0.663174	0.8812	0.4009	0.86573	0.7577
1%PE	2.1514	0.982	2.029084	1.045806	0.9879	0.140606	16.97793	0.9908
0.10%PLA	1.9982	0.9624	1.335058	0.868583	0.9827	0.049806	2.439024	0.9895
0.50%PLA	2.8586	0.9441	2.626637	1.156203	0.9202	0.469734	8.591065	0.9403
1%	3.0619	0.9564	2.967563	1.170001	0.9419	0.504664	9.242144	0.9486

Table S7. Bio-soil accumulation factor (BSAF) of boscalid in earthworms.

Time(d)	BOS		BOS+PE-MPs		BOS+PLA-MPs	
	BSAF Average	Standard Deviation	BSAF Average	Standard Deviation	BSAF Average	Standard Deviation
1	0.166428	0.228978**	0.228978**	0.025341	0.054054**	0.015661
3	0.187861	0.159912	0.159912	0.015819	0.072263**	0.033158
5	0.170562	0.172103	0.172103	0.021188	0.093789**	0.026795
7	0.182166	0.171184	0.171184	0.017845	0.167082	0.047119
14	0.207743	0.226315	0.226315	0.048945	0.421973**	0.042313
21	0.36925	0.382305	0.382305	0.012984	0.198948**	0.048862
28	0.267619	0.363566**	0.363566**	0.050922	0.155705**	0.019721

Note: Asterisks indicated that $p < 0.05$ compared with the BOS treatment group.

Table S8. PLS-DA model validation parameters and permutation testing results.

Comparison	R^2X	R^2Y	Q^2	Permutations	Permutation result	Overfitting assessment
CK vs BOS	0.622	0.874	0.757	200	R^2 intercept = 0.33, Q^2 intercept = -0.0786	No obvious overfitting
CK vs PE	0.592	0.931	0.808	200	R^2 intercept = 0.732, Q^2 intercept = -0.257	No obvious overfitting
CK vs BOS+PE	0.584	0.922	0.575	200	R^2 intercept = 0.402, Q^2 intercept = -0.0412	No obvious overfitting
CK vs PLA	0.248	0.934	0.719	200	R^2 intercept = 0.231, Q^2 intercept = -0.171	No obvious overfitting
CK vs BOS+PLA	0.738	0.906	0.638	200	R^2 intercept = 0.586, Q^2 intercept = -0.19	No obvious overfitting

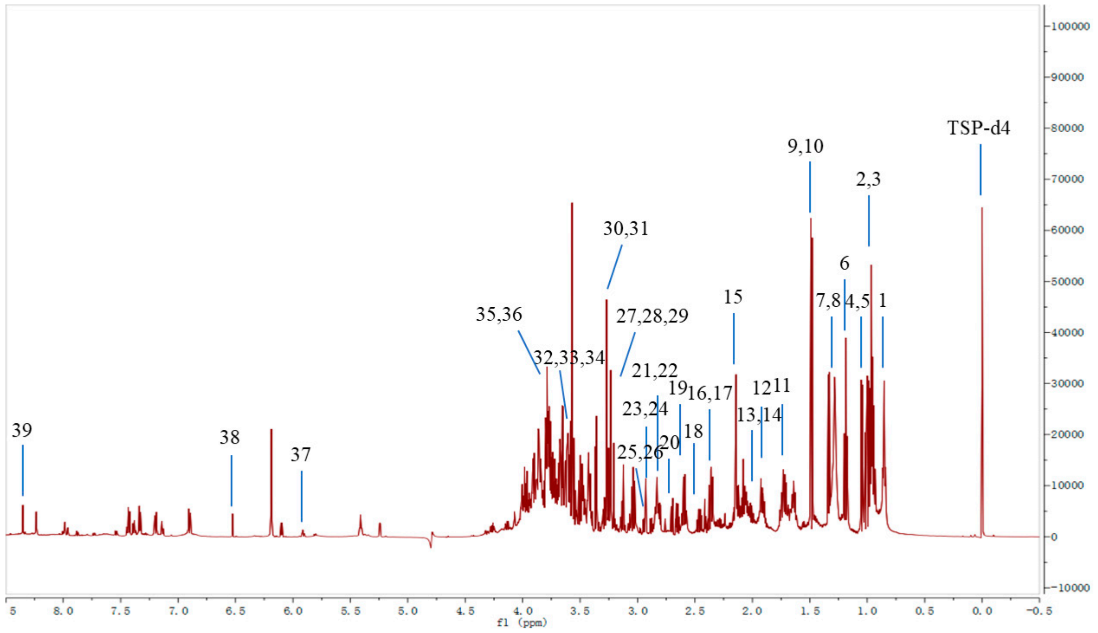

Figure S1. Representative 600MHz ^{1}H NMR spectra of earthworm samples.

1. Cholesterol; 2. Butyric acid; 3, leucine; 4, valine; 5, 3-hydroxyisobutyric acid; 6, 3-hydroxybutyric acid; 7. 3-hydroxyisovaleric acid; 8. Lactic acid; 9. Lysine; 10. Alanine; 11. Arginine; 12. Acetic acid; 13. Glutamic acid; 14. Glutamine; 15, methionine; 16, maleic acid; 17, pyruvate; 18 succinic acid; 19, citric acid; 20. Aspartic acid; 21. Sarcosine; 22, methyl guanidine; 23. Asparagine; 24, dimethylglycine; 25, creatine; 26, inosine; 27, cisaconite acid; 28, phenylalanine; 29. Choline; 30. Phosphatidyl ethanolamine; 31, taurine; 32, glucose; 33, glycine; 34, glycogen; 35, tyrosine; 36. Cysteine; 37, uracil; 38, fumaric acid; 39. Formic acid.

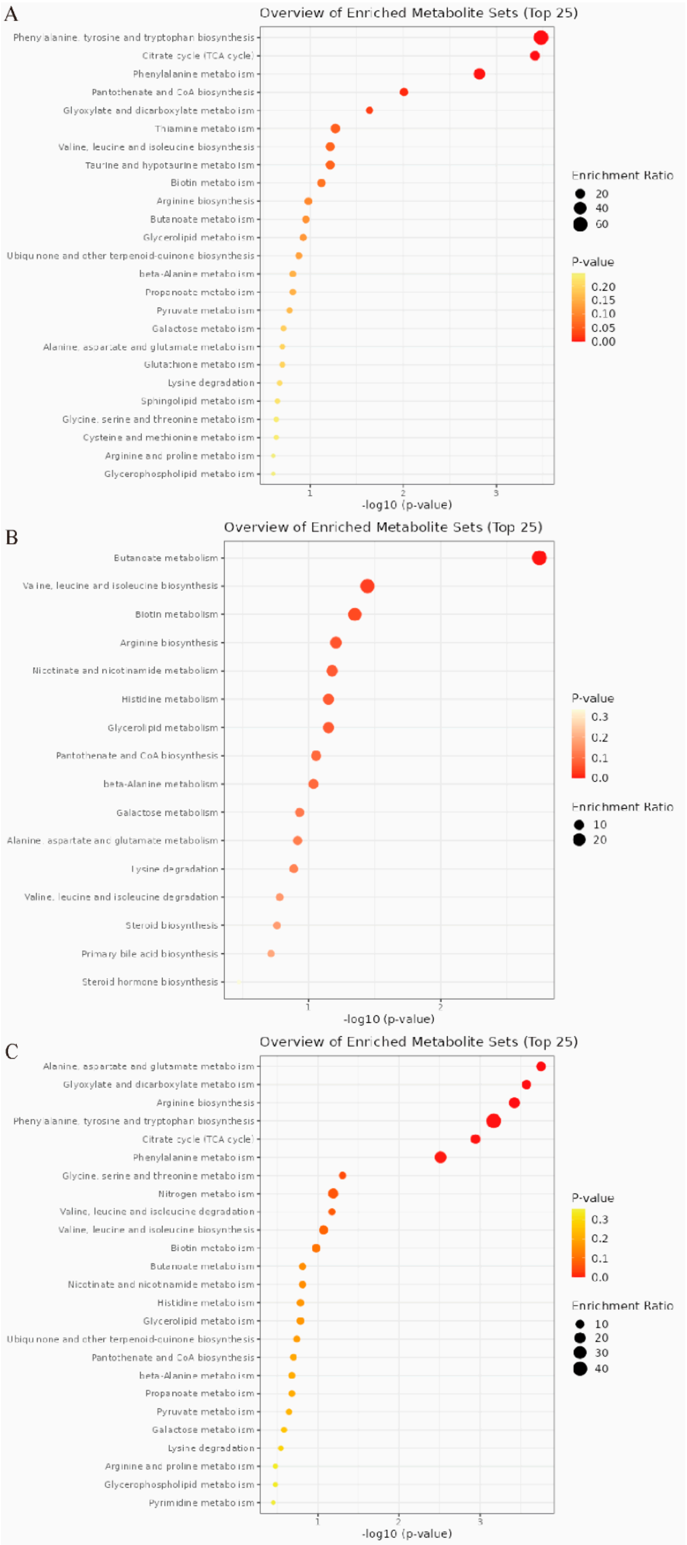

Figure S2. Enrichment of metabolic pathways of differential metabolites in earthworms

(A) BOS group; (B) PE group; (C) BOS+PE group

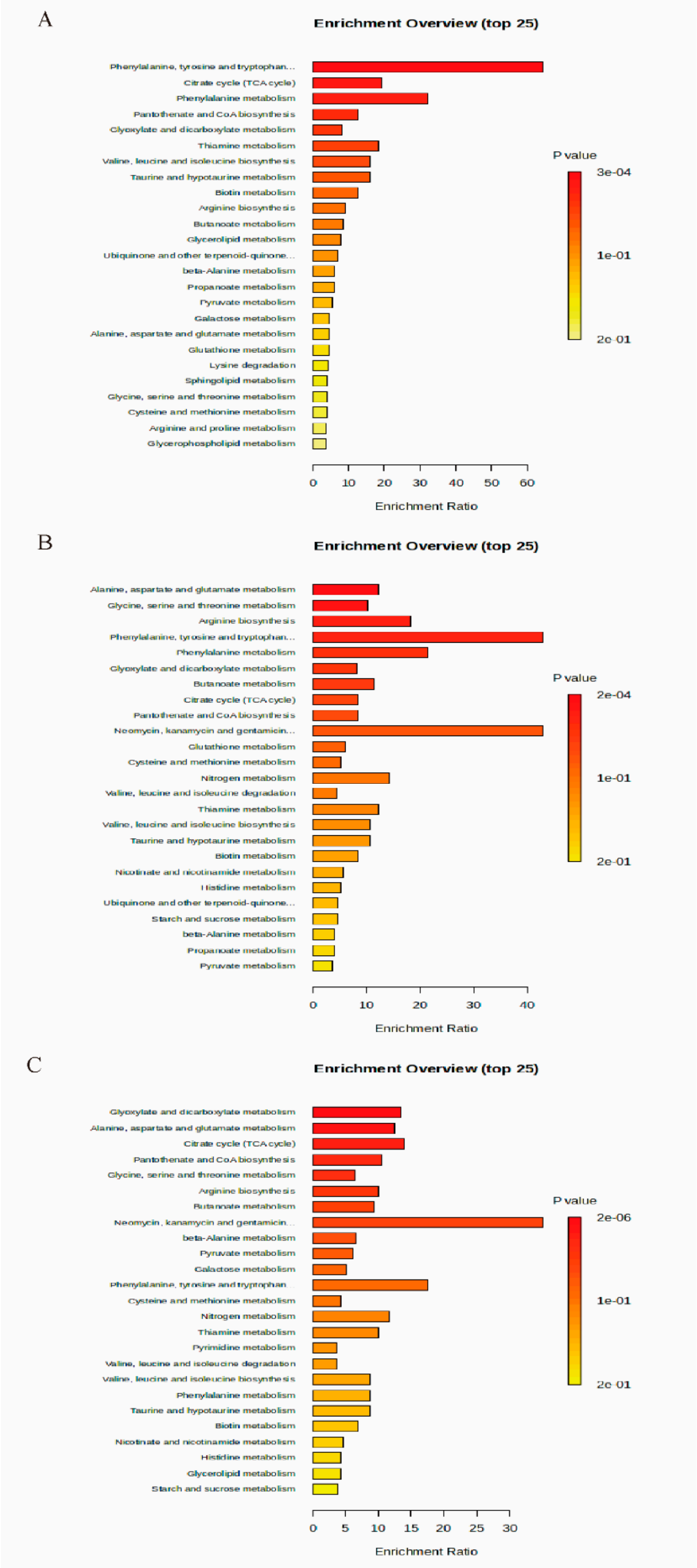

Figure S3. Enrichment of metabolic pathways of differential metabolites in earthworms

(A) BOS group; (B) PLA group; (C) BOS+PLA group
